# Not Your Typical Angioma

**Published:** 2015-01-12

**Authors:** Jenika S. Karcich, Alexis L. Parcells, Ramazi O. Datiashvili

**Affiliations:** Division of Plastic Surgery, Department of Surgery, Rutgers New Jersey Medical School Newark, NJ

**Keywords:** Lymphangioma, thumb angioma, lymphangioma thumb, lymphangioma hand, hand tumor

## DESCRIPTION

A 14-month-old female patient presents with a progressively enlarging right thumb mass. Her mother states that the mass developed shortly after birth. The patient had tenderness on palpation of the lesion, and she was noted to have difficulty grasping objects.

## QUESTIONS

1. **What important history and clinical examination findings differentiate this lesion from similar ones?**
2. **How is this lesion diagnosed?**
3. **What are the treatment options?**
4. **What is the risk of malignant transformation?**

## DISCUSSION

**Figure 1:**
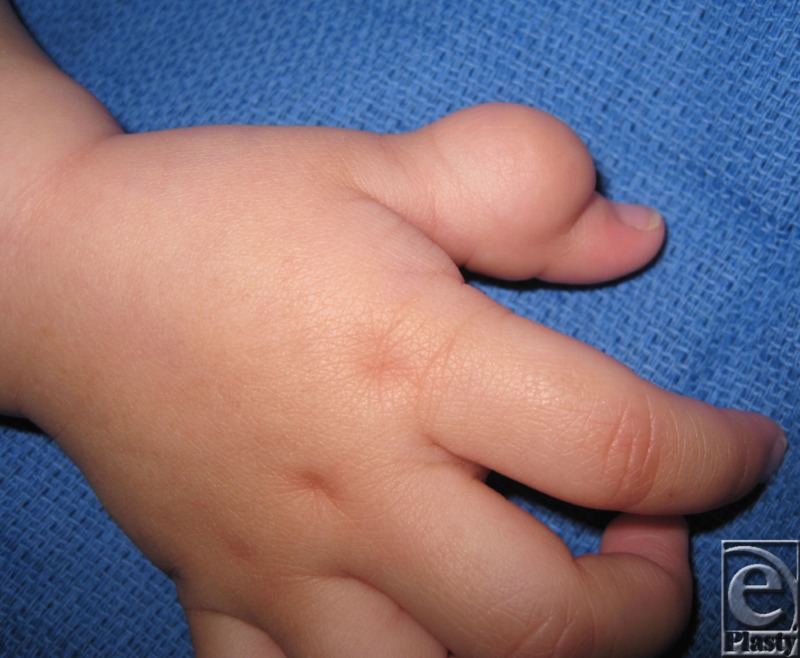
Thumb mass

Lymphangiomas are lymphatic malformations that result from failure of lymphatic channels to connect to their central drainage system, leading to poor lymph flow and fluid accumulation.^1^^,^^2^ Patients clinically present with compressible thin-walled vesicles ranging from clear to deep purple pigmentation.^1^^,^^3^ The lesions often compromise hand function (Fig 1).

**Figure 2:**
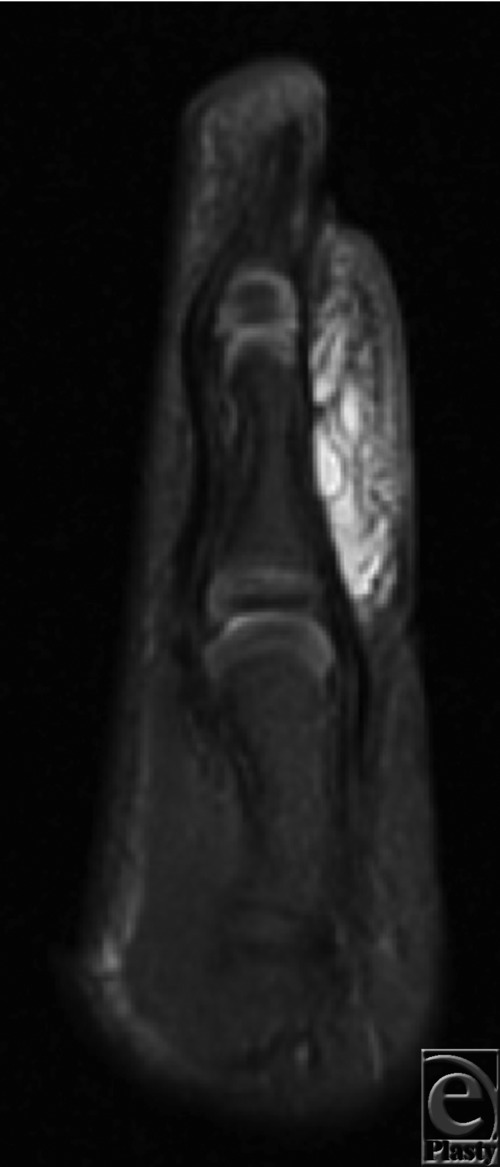
Magnetic resonance image of thumb mass.

Presentation of these masses is vague and may be easily confused with hemangiomas or venous malformations. Magnetic resonance imaging is useful in demonstrating characteristic lymphatic septa and venous channels that display low signal on T1 and high signal on T2 images^4^ (Fig 2). Biopsy is diagnostic, with histopathology showing dilated lymph vessels.^5^ Lymphangiomas are further classified as either microcystic (<2 cm^2^) or macrocystic (>2 cm^2^).

**Figure 3:**
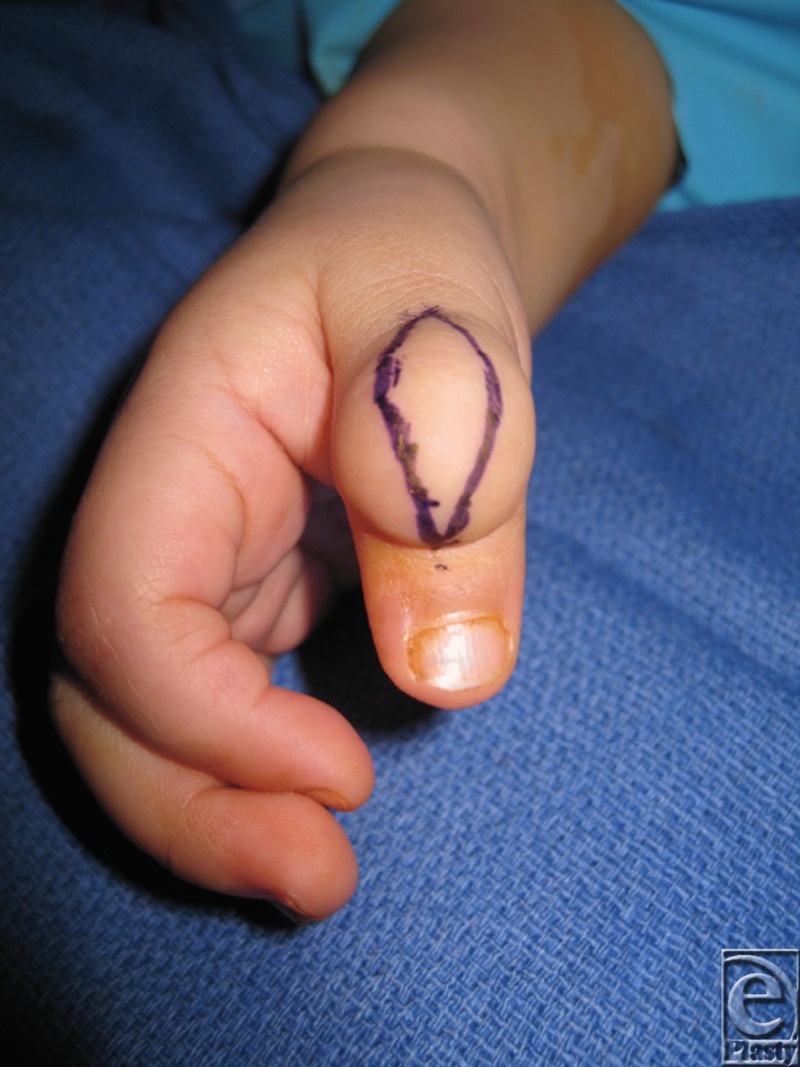
Preoperative marking of thumb mass.

**Figure 4:**
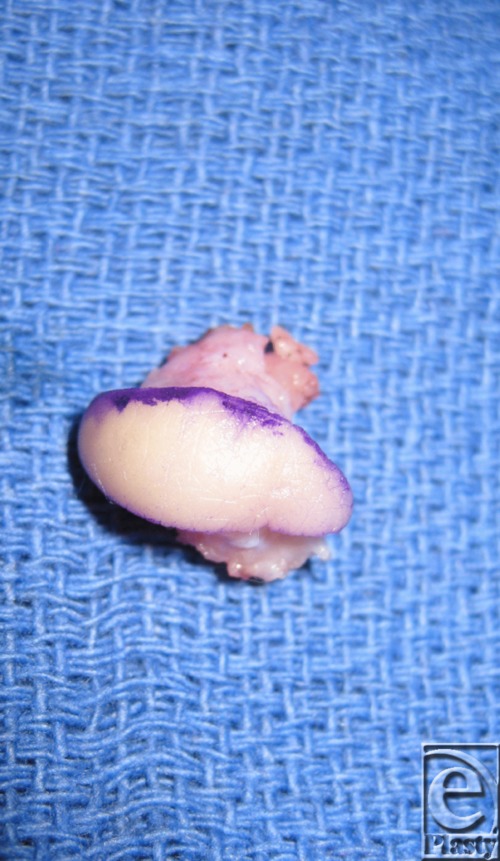
Postoperative excision of thumb mass.

Treatment of lymphangiomas is challenging, and current treatment regimens are suboptimal. A multidisciplinary approach can be invaluable to coordinating the best care possible for these patients. Noninvasive techniques such as external compression and observation are ineffective in limiting growth.^2^ Sclerotherapeutic agents including OK-432 and bleomycin have shown to be efficacious in clinical trials for macrocytic lesions (>2 cm), though neither is currently approved for use in the United Stats. Carbon dioxide and Nd:YAG lasers have shown efficacy in decreasing hemorrhage, postoperative pain, and recurrence.^6^ However, surgical treatment remains the best option for extensive disease in functional or cosmetically sensitive areas. The goals of surgery are to preserve function and improve appearance (Fig 3). Deep and wide excisions are often required to decrease risk of recurrence^7^ (Fig 4). Regardless of treatment, complete cure is unlikely and recurrence rates are still high. These patients must be closely monitored.

Lymphangiomas present virtually no risk of malignant transformation.^7^ However, cases of lymphangiosarcoma secondary to chronic lymphedema of an extremity have been documented.

Lymphangioma of the hand is a rare tumor with a vague presentation. Magnetic resonance imaging is an important diagnostic modality. These cases are challenging, and current treatment options to reduce size include sclerosing agents, laser embolization, and surgical excision. Our patient underwent surgical excision with wide margins. Patients must be followed closely for recurrence. Risk of malignant transformation is low.
